# Crystalloid and Colloid Compositions and Their Impact

**DOI:** 10.3389/fvets.2021.639848

**Published:** 2021-03-31

**Authors:** Elke Rudloff, Kate Hopper

**Affiliations:** ^1^BluePearl Specialty + Pet Emergency, Glendale, WI, United States; ^2^Department of Veterinary Surgical & Radiological Sciences, School of Veterinary Medicine, University of California, Davis, Davis, CA, United States

**Keywords:** crystalloid, colloid, plasma, osmolarity, fluid therapy, COP, SID, strong ion difference

## Abstract

This manuscript will review crystalloid (hypo-, iso-, and hyper-tonic) and colloid (synthetic and natural) fluids that are available for intravenous administration with a focus on their electrolyte, acid-base, colligative, and rheological effects as they relate to each solution's efficacy and safety. The goal is for the reader to better understand the differences between each fluid and the influence on plasma composition, key organ systems, and their implications when used therapeutically in animals with critical illness.

## Introduction

Water is the body's universal solvent and the most essential nutrient of the body. Within the vessel, water is the transport medium that brings oxygen, solutes, and hormones to the interstitium and removes waste products for breakdown and excretion. Within the interstitial space, water allows movement of these substances between the capillary and the cell. Within the cell, water provides a medium for organelles that gives the cell its form. Water also functions to dissipate heat through evaporation.

Restoring and maintaining fluid balance in the critically ill or injured animal can be one of the most difficult challenges of patient management. Intrinsic factors that dictate the movement of crystalloid fluids into individual body fluid compartments (intracellular, interstitial, intravascular) include the normal distribution of total body water, and the factors involved with the movement of fluids across the capillary membrane (e.g., transmembrane hydrostatic, colloid osmotic pressure, and the endothelial glycocalyx and endothelial membrane integrity), as well as cellular membrane and lymphatic function (1, 2). Fluid composition and volume infused can impact hydrostatic pressure, colloid osmotic pressure (COP) and extracellular fluid osmolality. These changes will ultimately determine the volume kinetics of distribution (3). An understanding of the fluid types and their unique properties is necessary to ensure that therapy will have the desired effect on the fluid compartment being targeted, and minimize complications associated with fluid therapy.

## Fluid Types

### Crystalloid Solutions

A crystalloid solution is an aqueous solution composed of water and small solutes such as electrolytes and glucose (4, 5). Crystalloid solutions can be categorized based on whether they are hypotonic, isotonic, or hypertonic (Table 1). Tonicity describes the effective osmolality of a fluid, which is the ability of a fluid to alter water movement across the cell membrane. It is important to note that fluid osmolality may not equate to tonicity; for example, a fluid can be iso-osmolar and yet hypotonic (see discussion below).

**Table 1:** Characteristics of commonly used intravenous fluids.

**NAME**	OSMOLARITY* (mOsm/L)	**pH**	**Na+ (mEq/L)**	**Cl- (mEq/L)**	**K+ (mEq/L)**	**Mg++ (mEq/L)**	**Ca++ (mEq/L)**	**Dextrose (g/L)**	**Organic anion (mEq/L)**	**COP (mmHg)**	**SID (mEq/L)**
**CRYSTALLOID**											
**Hypertonic**											
7.2% saline	2,396		1,197	1,197	0	0	0	0	0	0	0
**Isotonic**											
0.9% Saline	308	5.0	154	154	0	0	0	0	0	0	0
Lactated Ringer's	275	6.5	130	109	4	0	3	0	Lactate (28)	0	27
Plasma Lyte-A®	294	7.4	140	98	5	3	0	0	Acetate (27), Gluconate (23)	0	27–50
Plasma Lyte-148® or Normosol-R®	294	5.5	140	98	5	3	0	0	Acetate (27), Gluconate (23)	0	27–50
Sterofundin®-Iso	309	5.5–5.9	145	127	4	2	5	0	Acetate (24), Malate (5)	0	25.5
Hypotonic**											
Plasma Lyte-56 in 5% dextrose®	363	3.5–6	40	40	13	3	0	5	Acetate (16)	0	16
0.45% Sodium chloride + 2.5% dextrose	280	4.5	77	77	0	0	0	25	0	0	0
5% Dextrose in water	252	4.0	0	0	0	0	0	50	0	0	0
0.45% Sodium chloride	154	5.6	77	77	0	0	0	0	0	0	0
**COLLOID**											
**Natural**											
Whole blood	300	Variable	140	100	4	0	0	0–4	0	20	
Frozen plasma	300	Variable	140	110	4	0	0	0–4	0	20	12
25% Human serum albumin			0	0	0	0	0	0	0	200	
5g Lyophilized canine albumin			0	0	0	0	0	0	0		
**Synthetic**											
6% Hetastarch 450/0.7 Hespan™	310	5.5	154	154	0	0	0	0	0	26	0
6% Hetastarch 450/0.7 Hextend™	304	5.9	143	124	3	0.9	5	0.99	Lactate (28)	31	28
6% Pentastarch 200/0.5 HAES-steril 6%™	308	5.0	154	154	0	0	0	0	0	30–35	0
10% Pentastarch 200/0.5 HAES-steril10%™	308	5.0	154	154	0	0	0	0	0	25	0
6% Tetrastarch 130/0.4 VetStarch™ Voluven™	308	4–5.5	154	154	0	0	0	0	0	37	0
6% Tetrastarch 130/0.42 Tetraspan™	296	5.6–6.4	140	118	4	1	2.5	0	Acetate (24), Malate (5)	40	

*The value provided is the calculated osmolarity which may overestimate the measured osmolality of the fluid. The osmolarity of a solution is relevant to the impact on erythrocytes on infusion while the effective osmolality (tonicity) will dictate the volume of distribution of the fluid.

**These solutions are either hypotonic at the time of infusion, or become so when the dextrose is metabolized.

*COP, Colloid osmotic pressure; SID, Strong ion difference in vivo*.

#### Hypotonic Crystalloids

A hypotonic fluid has an effective osmolality significantly lower than that of the patient. Infusion will reduce the osmolality of the extracellular fluid resulting in redistribution of water into the intracellular compartment. Dextrose is often added to hypotonic fluids so that they are isosmotic during intravenous administration to prevent hemolysis. However, as the dextrose moves into cells and is metabolized, the fluid becomes hypotonic and they are expected to redistribute to the intracellular compartment based on total body water distribution, i.e., ~66% of the infused volume of solute-free water will move into the intracellular space.

Hypotonic crystalloid solutions are used for sustaining maintenance fluid requirements, treatment of solute-free water deficits, and drug administration. Maintenance-type crystalloid fluids replace ongoing sensible (measurable; urine, feces, sweat) and insensible (unmeasurable; transepidermal diffusion/evaporation, respiratory evaporation, fever) water lost because of basic bodily functions (6). Solute-free water is lost with insensible losses, and therefore more water than solutes are needed for maintaining fluid balance. Maintenance-type crystalloids have a sodium concentration in the range of 40–77 mEq/L and can contain additional anions and cations (Table 1). The osmolality of a maintenance fluid will vary depending on its constituents. The addition of dextrose can make a hypotonic fluid isosmolar at the time of administration. The distribution of maintenance type fluids will expand the intracellular, interstitial and to a minimal extent, the intravascular space. The degree of intracellular vs. extracellular distribution will be dictated by the effective osmolality (tonicity) of the fluid administered.

Dextrose containing solutions that are otherwise solute-free (i.e., do not contain sodium) can be hypo-, iso- or hyper-osmolar depending on the concentration of dextrose in the fluid. They become hypotonic as the dextrose is metabolized leaving solute-free (pure) water. An isosmotic fluid, 5% dextrose in water (D5W) is a source of solute-free water that can be used to treat a solute-free water deficit or as a diluent for drug administration. Although D5W can be used in the treatment of hypoglycemia, caution is needed to avoid iatrogenic hyponatremia. Hyperosmolar dextrose solutions containing 25–50% dextrose can be administered alone (ideally through a central venous line to avoid phlebitis) or added to an isotonic crystalloid solution to provide dextrose supplementation without the risks of hyponatremia.

#### Isotonic Crystalloids

An isotonic crystalloid fluid has an effective osmolality like the patient. The effective osmolality of common isotonic fluids ranges from 270 to 310 mOsm/L. Isotonic fluids have a similar sodium concentration as the extracellular fluid compartment and have minimal impact on intracellular volume. Isotonic crystalloid fluids can vary in their concentration of the electrolytes sodium, chloride, potassium, magnesium, and calcium (Table 1). They may also contain organic anions such as lactate, gluconate, and acetate. The electrolytes and organic anions will contribute to a solutions strong ion difference (SID), and can impact the pH following metabolism of the organic anions (5, 7), except for gluconate, which is mostly excreted unchanged in the kidneys.

A crystalloid fluid is considered balanced if it contains electrolytes in similar concentration to the individual's plasma, maintains or normalizes acid-base balance through its SID, and is iso-osmotic and isotonic to the patient's normal plasma (5, 7). With that definition, there may be few true balanced crystalloid solutions available, since each species and particular patient may vary in their normal plasma make-up. As a fluid is infused, the *in vivo* SID will impact the patient's SID resulting in alteration in the patient's acid-base status. The solution 0.9% sodium chloride is the most unbalanced isotonic crystalloid, given its high, un-physiologic, chloride concentration and its 0 value SID (5, 7). For the purposes of this article, “balanced” isotonic crystalloids will be referencing those traditionally named as such, i.e., Plasma-Lyte 148 and lactated Ringer's solution (LRS).

Isotonic crystalloids are used to replenish extracellular fluid deficits, and as discussed below, to maintain extracellular fluid volume. The volume kinetics of isotonic crystalloids is variable and influenced by the rate of infusion, patient's physiological condition, degree of dehydration, surgery, and anesthesia (see section on volume kinetics) (8, 9). This distribution will be altered with changes in COP, vascular permeability, and changes in the extracellular matrix. Up to 50% of the intravascular volume effect can be lost in as little as 30 min in people with normal transcapillary fluid dynamics (i.e., no hypotension, altered capillary permeability, etc.) (8). The volume of distribution of isotonic crystalloids between the intravascular and interstitial space in health will approximate the relative size of each compartment i.e., ~25% will remain intravascular, 75% will remain interstitial. Examples of conditions where an isotonic crystalloid is used most effectively include dehydration, hemorrhage, vomiting, diarrhea, and effusive diseases.

#### Hypertonic Crystalloids

A hypertonic fluid is a fluid that has an effective osmolality (tonicity) greater than that of the patient. Infusion will increase the osmolality of the extracellular fluid resulting in redistribution of water out of the intracellular fluid compartment, increasing extracellular volume. Common hypertonic solutions used in veterinary medicine include hypertonic saline (HTS) and mannitol. The volume effects of these solutions are short lived, since the small solutes will be redistributed and/or excreted.

Hypertonic saline is available at various concentrations, with 3%, 7.2–7.5%, and 23.4% being commonly available. The 23.4% HTS is not recommended for direct infusion and should be diluted before administration. Hypertonic saline in the 3–7.5% range can be infused without dilution or in combination with isotonic crystalloids and/or colloids for hypovolemic shock resuscitation.

Hypertonic saline is also used for customizing crystalloid fluid solutions to target a desired sodium concentration, most commonly for the treatment of patients with dysnatremia. In addition, mannitol and HTS can be prescribed for patients with signs of intracranial hypertension. Hypertonic saline effects include increasing plasma osmolality and sodium and chloride concentration, endogenous release of vasopressin, and immunomodulation (10–13). The impact of infusing a supraphysiologic dose of chloride should be considered, although a direct link between HTS and risk of acute renal injury has not been established (10).

### Colloid Solutions

A colloid solution contains large molecular weight particles such as proteins or hydroxyethyl starches (HES) suspended in a crystalloid solution (4). The large insoluble molecules do not easily cross the endothelial glycocalyx and membrane. The force exerted on the endothelial surface layer due to the osmotic gradient created by these proteins is called COP or oncotic pressure. Colloid solutions can be divided into natural and synthetic colloids.

#### Natural Colloids

Natural colloids are protein-containing products, such as whole blood, plasma, and concentrated albumin solutions. Although any protein can contribute to COP, albumin (~67,000 Daltons), is most relevant because it is the smallest and most numerous of the protein particles, and, the overall negative charge of albumin attracts positive sodium molecules into its orbit, thereby increasing its osmotic capability by ~20% (the Gibbs-Donnan effect) (14–16). Albumin also has antioxidant properties, scavenges oxygen-free radicals, and is a carrier protein for steroids, drugs, bilirubin, fatty acids, and hormones (15, 17).

Plasma can be stored as fresh frozen plasma (FFP), frozen plasma, liquid (refrigerated) plasma, cryosupernatant (cryopoor), or stored whole blood. The concentration of albumin in these products will depend on the albumin concentration of the donor animal and is ~2.5–3 g/dL (18). Of the plasma products, cryoprecipitate does not contain albumin. The choice of the optimal plasma product for a patient will depend on other patient factors such as coagulation status as well as product availability. The volume of plasma products needed to increase albumin levels and provide effective COP support often exceeds product availability or is cost prohibitive. Calculation of albumin deficit demonstrates that ~45 ml/kg of plasma is required to increase serum albumin by 1 g/dL, assuming there are no ongoing albumin losses.

Lyophilized canine albumin is stored in a dehydrated powder form and reconstituted with 0.9% sodium chloride or 5% dextrose in water to a desired albumin concentration (5–16%). Infusion of a 16% solution (16 g/dL albumin) results in an ~1.2 times volume expansion. It seems well-tolerated, can increase serum albumin concentrations in dogs with septic peritonitis, and can increase albumin and COP in healthy dogs (19, 20).

Human serum albumin (HSA) concentrates are reconstituted to contain 5–25% albumin. The high concentration of albumin and resulting high COP (200 mmHg) in 25% HSA (25 g/dL albumin) has the greatest capability of increasing plasma albumin and COP (21), however acute and delayed hypersensitivity reactions have been reported in dogs, which can be fatal (18, 22, 23). Infusion of HSA has been done in critically ill dogs and cats to increase serum albumin without reports of hypersensitivity reactions (24, 25).

To estimate the volume of a transfusion product required to increase serum albumin the following formula can be used (26).

Albumin deficit (g) = 10 [0.3 × body weight (kg) × (target albumin concentration (g/dL) − patient albumin concentration (g/dL))].

#### Synthetic Colloids

Synthetic colloids include gelatins, starches, dextrans and complex polysaccharides. Gelatins and dextrans are infrequently used. Hydroxyethyl starch is one of the most common type of synthetic colloid in use in veterinary medicine. It is a synthetic polymer of glucose (98% amylopectin), made from a waxy species of plant starch such as maize, potatoes or sorghum (27). It is a highly branched hydrophilic polysaccharide closely resembling glycogen, formed by the reaction between ethylene oxide and amylopectin in the presence of an alkaline catalyst. The HES product varies in molecular characteristics and these will determine the clinical effects including half-life, coagulation effects, impact on COP and volume expanding effects. The main characteristics of HES that are relevant are the concentration, molecular weight (MW), molar substitution, and C2:C6 ratio (Table 2) (27, 28). The concentration influences the COP and hence the volume expanding effects.

**Table 2:** Characteristics of commonly used hydroxyethyl starch products.

**Hydroxyethyl starch product**	**Commercial name**	**Average molecular weight (kDa)**	**Molar substitution**	**C2:C6 ratio**	**COP (mmHg)**
6% Hetastarch 450/0.7	Hespan™	450	0.7	4.5:1	26
6% Hetastarch 450/0.7	Hextend™	450	0.7	4.6:1	31
6% Pentastarch 200/0.5	HAES-steril 6%™	200	0.4–0.5	5:1	30
10% Pentastarch 200/0.5	HAES-steril10%™	200	0.4–0.5	5:1	66
6% Tetrastarch 130/0.4	VetStarch™; Voluven™	130	0.4	9:1	36

Hydroxyethyl starch contains a polydisperse number of molecules with different MW. The MW impacts the osmotic pressure and the half-life of the product as well as the coagulation effects. Small MW molecules that are below the renal threshold (<70 kDa) will be renally excreted and the intravascular expanding effects are quickly lost. Higher MW molecules have greater impact on coagulation, and this will limit the safe daily dose of the product. Tetrastarches have a medium MW and have been shown to have less coagulation effects in comparison to an equal dose of the high MW hetastarch products (29), although at low doses this may not be clinically relevant (30).

The molar substitution is the calculated average number of hydroxyethyl groups per glucose residue in the molecule. It can be adjusted by the degree of substitution of hydroxyl groups with hydroxyethyl groups at the C2, C3, and C6 positions on the glucose molecule (27). The C2:C6 ratio indicates the degree of substitution of hydroxyethyl groups on position C2 in relation to C6 of the glucose molecule. The higher the molar substitution and the greater the C2:C6 ratio, the slower the degradation of the molecule by amylase. For example, hetastarch 450/0.7 has an average MW of 450 kDa and molar substitution of 0.7 (hence “heta”) and has a longer half-life than tetrastarch 130/0.4, which has an average MW of 130 kDa and a molar substitution of 0.4 (hence “tetra”). The HES products are suspended in an isotonic crystalloid solution that vary in constituents as described in **Tables 1, 2**.

Degree of volume expansion and longevity of HES, or any colloid, in plasma is difficult to predict. It will depend not only on the degree of vascular injury, but also on the MW and dispersity, amylase concentration, elimination rate into the urine, as well as its electrical charge, shape, and effect on the endothelial glycocalyx (31–37). Studies focusing on the immunologic, coagulation, and renal effects of HES products are not all in agreement, and species differences may exist.

## Fluid Choice

Despite IV fluids being commonly prescribed medications in human and veterinary medicine, the optimal fluid choice remains poorly defined. There is growing evidence in human medicine that fluid choice can have an impact on outcome, particularly in critically ill patients. Specific recommendations for the single best type of crystalloid or colloid fluid to infuse cannot be made based on current information. Patient factors to be considered when selecting an IV fluid type include volume of administration, the patient's electrolyte and acid-base status, and ongoing disease processes. Fluid factors to be considered include tonicity, electrolyte and organic anion concentration, and compatibility with drugs or other fluids that will be co-administered.

### Replacement vs. Maintenance Fluids

Maintenance-type fluids were designed to replace solute-free water and electrolyte losses in the otherwise healthy, fasting patient. The composition of maintenance fluids was determined from research performed in healthy children, and the electrolyte composition was determined to resemble the electrolyte composition of milk (38, 39). The water and electrolyte requirements of healthy dogs and cats is described by the National Research Council and these differ greatly from the composition of commercially available fluids (40, 41).

It has been advocated that isotonic crystalloids are given to replenish any extracellular fluid deficits and replace ongoing losses while maintenance type (hypotonic) fluids are given for daily maintenance needs. This requires two fluid types which can increase cost, and clinical experience has shown that most patients can be adequately managed with an isotonic crystalloid alone. Maintenance-type fluid therapy alone may be considered in the patient that has adequate intravascular and interstitial fluid volumes without ongoing extracellular volume losses. An example may be the cat that sustained a jaw fracture and is unable to drink water. Maintenance type fluids may also be considered in the patient that is developing hypernatremia, although adequate support of extravascular volume should be monitored. As maintenance type fluids have less effect on extracellular fluid volume than isotonic crystalloids, they have been favored in patients at risk of volume overload, such as animals with heart disease or kidney disease.

Careful consideration should be taken when prescribing maintenance type fluids for the treatment of sick patients that have extracellular fluid deficits and/or ongoing abnormal losses. The major adverse events that may be associated with the administration of maintenance type fluids are ineffective intravascular volume support and hyponatremia. Acute, hospital acquired hyponatremia has been associated with the administration of hypotonic, maintenance type fluids in human patients (39, 42, 43). Randomized controlled trials comparing isotonic crystalloids with hypotonic crystalloids as ongoing maintenance fluid therapy in adult humans and pediatric patients have shown isotonic crystalloids to be safe and is associated with a lower incidence of hyponatremia (44–47).

### Sodium Concentration

As Table 1 shows, there is a wide range of sodium concentration in the commonly used IV fluids. For most clinical scenarios, balanced isotonic crystalloids (e.g., LRS, Plasma-Lyte 148) are recommended. Although these fluids have a slightly lower sodium concentration than normal dogs and cats, they are considered isotonic in clinical practice. However, animals suffering intracranial hypertension may benefit from receiving an isotonic fluid with a higher sodium concentration (e.g., 0.9% sodium chloride).

When treating animals with an abnormal sodium concentration the general guideline is to avoid changing the sodium concentration rapidly (48). When administering large volumes of fluids rapidly to animals with a significant dysnatremia, it is recommended to use a fluid with a sodium concentration within ~10 mEq/L of the patient. It is generally considered safe for patients with a sodium concentration up to 165–170 mEq/L to receive 0.9% sodium chloride. If a patient with a sodium concentration >170 mEq/L requires rapid administration of large volumes of fluid, it may be best to make fluid prescription isotonic to the patient by the addition of hypertonic saline.

Lactated Ringer's solution is an ideal fluid for patients with hyponatremia unless they have a sodium <120 mEq/L. In that situation, if large volumes of fluids are indicated, it may be necessary to further dilute the sodium concentration with water to make a fluid prescription that is isotonic to the patient.

### Chloride Concentration

Balanced isotonic crystalloid solutions have a chloride concentration similar or slightly less than healthy dogs and cats while 0.9% sodium chloride and HES in 0.9% sodium chloride have a higher chloride concentration than normal dogs and cats. Human medicine has traditionally used 0.9% sodium chloride rather than balanced isotonic crystalloids for fluid therapy, and in more recent years the incidence and potential adverse effects of hyperchloremia has been a focus of many investigations.

Chloride has several important physiological roles including regulation of glomerular filtration rate, blood pressure responses, gastrointestinal function, and acid-base homeostasis. The impact of hyperchloremia was demonstrated by a study in a human ICU that evaluated the effect of changing fluid therapy from a chloride-liberal approach (infusing 0.9% sodium chloride or colloids suspended in 0.9% sodium chloride) to a chloride-restricted approach (infusing Hartmann's, Plasma-Lyte 148, etc.) (49). The incidence of hyperchloremia and metabolic acidosis was significantly decreased with the chloride-restricted fluid approach. In a randomized clinical trial, the administration of balanced crystalloids resulted in a lower mortality rate and lower rates of renal replacement therapy or renal dysfunction in critically ill patients, when compared to saline administration (50). In comparison, no difference in outcome could be demonstrated in a similar study of non-critically ill adults (51). In a large veterinary study of all dogs and cats with a serum chloride measured, hyperchloremia was identified in 21% of dogs and 9% of cats and was associated with a higher mortality than those with normochloremia (52).

Potential mechanisms for the harmful effects of administration of chloride rich fluids include altered cytokine responses, increased nitric oxide levels, hemodynamic instability and altered renal blood flow (53). Hyperchloremic metabolic acidosis has been shown to decrease renal blood flow and some studies have found an association with acute kidney injury. Rapid administration of 2 L 0.9% sodium chloride to healthy human volunteers caused a significant reduction in renal arterial blood flow and renal cortical tissue perfusion, when compared to baseline and to the people who received Plasma-Lyte 148 (54). An experimental dog study demonstrated that renal vasoconstriction is a direct result of hyperchloremia (55). Increased renal tubular chloride concentration has been shown to cause increased absorption of chloride by the macula densa leading to afferent arteriolar vasoconstriction. Fluid retention in the interstitial space is also greater with 0.9% sodium chloride than with balanced isotonic solutions. This may further limit urine output and contribute to organ dysfunction. Despite these concerns, the relationship between hyperchloremic metabolic acidosis and kidney injury is not clear and there appears to be a difference in the impact of hyperchloremia in the critically ill patient population. The exact relationship between hyperchloremia and outcome remains to be determined but avoiding iatrogenic hyperchloremia is a strong clinical recommendation at this time.

Hypochloremic metabolic alkalosis is not uncommon and is usually the result of selective gastric loss of hydrochloric acid from abnormalities such as pyloric outflow obstruction. In this case, 0.9% sodium chloride is the ideal fluid therapy as it will increase serum chloride concentration effectively and promote bicarbonate excretion by the kidney to enhance the normalization of acid-base balance.

### Potassium Concentration

Since 0.9% sodium chloride contains no potassium, it is commonly recommended for use in patients with hyperkalemia. Potassium homeostasis is impacted by extracellular sodium concentration as well as potassium concentration with sodium-potassium exchange having an important influence. The small quantity of potassium in balanced isotonic crystalloids such as LRS or Plasma-Lyte 148 is outweighed by the benefit of the lower sodium concentration when compared to 0.9% sodium chloride. Studies in human patients have shown that the administration of 0.9% sodium chloride in patients prone to hyperkalemia is associated with a similar or higher serum potassium concentration than in patients treated with balanced isotonic crystalloids, and other adverse effects, such as hyperchloremic metabolic acidosis, were common (56, 57). The administration of balanced isotonic crystalloids in animals with hypoadrenocorticism or urethral obstruction has been shown to provide more rapid correction of acid-base imbalances and may prevent neurological injury secondary to rapid correction of hyponatremia, with no significant differences found in time to potassium correction compared to 0.9% sodium chloride (58–61).

None of the commercially available isotonic crystalloids contain a substantial quantity of potassium and, as a result, addition of potassium to intravenous fluids may be necessary for patients receiving longer term fluid therapy. Potassium supplementation of IV fluids can be with potassium chloride or potassium acetate additives, either with or without potassium phosphate (Table 3). Potassium supplementation is ideally calculated according to serum potassium level and, unless life-threatening hypokalemia is occurring, the rate should not exceed 0.5 mEq/kg/h. The existing potassium in the administered fluid is taken into consideration when calculating a rate of replacement.

**Table 3:** Guideline for potassium supplementation for hypokalemia.

**Patient potassium**	**Rate of potassium administration**
Normal to mild hypokalemia (>3.5 mEq/L)	0.05–0.1 mEq/kg/h
Moderate hypokalemia (2.5 to 3.5 mEq/L)	0.3 mEq/kg/h
Severe hypokalemia (<2.5 mEq/L)	0.4–0.5 mEq/kg/h

### Magnesium Concentration

Hypomagnesemia occurs commonly in critically ill cats and dogs and magnesium supplementation can be challenging. Plasma-Lyte and Normosol fluids contain supplemental magnesium and may be used in patients with suspected hypomagnesemia, patients considered at risk of hypomagnesemia, and patients documented to have mild hypomagnesemia. A suggested slow replacement dose for magnesium is 0.3–0.5 mEq/kg/day (62). At maintenance rates (~3 ml/kg/h), Plasma-Lyte and Normosol solutions would provide 0.2 mEq/kg/day of magnesium. For patients documented to have moderate to severe hypomagnesemia additional magnesium supplementation is indicated. In patients with hypermagnesemia, it is suggested to avoid magnesium containing fluids. Magnesium containing fluids are compatible with blood products (63, 64).

### Calcium Concentration

Lactated Ringer's solution contains a small quantity of calcium (0.020 g/L CaCl_2_), which is insufficient to treat a patient with hypocalcemia. It has been commonly recommended to avoid co-administration of LRS with blood products due to concerns that the calcium could be chelated by the citrate anticoagulant in the blood product, leading to loss of citrate activity and clotting of the blood product. Several studies have shown that the calcium concentration in LRS is not associated with increased coagulation of blood products, and LRS is safe to co-administer with blood transfusions (65, 66).

### Acid-Base Effects of Fluids

#### pH of the Bag

An obvious difference between crystalloid fluid constituents and plasma is the difference in pH of the fluid itself (excluding Plasma-Lyte A). Crystalloid fluids tend to be acidic in nature. This is due the reaction of air with water and the process of heat sterilization. In addition, manufacturers often adjust the pH to a more acidic level for reasons such as improving stability and inhibition of microbial growth. The low pH of IV fluids has led to the suggestion that they can cause or worsen an acidemia. Although the pH of fluids can be low, the total quantity of acid (titratable acidity) is minimal and very readily buffered by the body. It will not significantly alter the acid-base balance of a patient (67). Plasma-Lyte A is essentially equivalent to Plasma-Lyte 148 or Normosol-R other than having a pH of 7.4. The total difference in hydrogen ion concentration between Plasma-Lyte 148 and Plasma-Lyte A is inconsequential to the body (68). An experimental study of hemorrhagic shock in pigs compared the acid base effects of Plasma-Lyte A, LRS and 0.9% sodium chloride and found no difference in the acid base balance of the animals resuscitated with Plasma-Lyte A or LRS (68).

#### Hyperchloremic Metabolic Acidosis

As previously discussed, the administration of chloride rich fluids (i.e., 0.9% sodium chloride) can cause hyperchloremia and a metabolic acidosis (49, 67). The delivery of high chloride filtrate to the kidney reduces renal bicarbonate reabsorption (69). The acid-base effect can also be considered in terms of the SID. Healthy dogs and cats have a SID of ~30–40 mEq/L (depending on laboratory reference ranges). Administration of fluids with a low SID will reduce the patient's SID, promoting a metabolic acidosis, whereas high SID fluids will promote a metabolic alkalosis. Chloride rich fluids have a low (or zero in the case of 0.9% sodium chloride) SID and as such are considered acidifying fluids. Since balanced isotonic crystalloids are used more commonly than 0.9% sodium chloride in veterinary medicine, hyperchloremic metabolic acidosis is less of a concern (70). The addition of potassium chloride to IV fluids can result in hyperchloremia and monitoring chloride in animals receiving fluid therapy for any sustained period is recommended.

#### IV Fluids Containing Organic Anions

An ideal isotonic crystalloid would be one that matches the composition of plasma. Plasma of normal dogs and cats generally has a sodium concentration of ~140–150 mEq/L, a chloride concentration of ~95–110 mEq/L, and a bicarbonate concentration of ~20 mEq/L. A crystalloid with this composition is appealing, however, bicarbonate is difficult to keep stable in solution and needs to be stored in glass. As a result, manufacturers have added organic anions to IV fluids that are stable in solution and will act as “bicarbonate precursors” or buffers. The metabolism of these substances in the body leads to the consumption of hydrogen ions (equivalent to the production of bicarbonate). Commonly used organic anions include lactate, acetate, and gluconate. Gluconate it excreted in the urine and not metabolized does not contribute to the acid base balance of the patient (71, 72).

Metabolism of organic anions such as lactate and acetate can increase plasma bicarbonate concentration in a dose dependent manner and can promote the development of a metabolic alkalosis (49, 71). The acid-base effect of organic anions can also be reflected by the fluid SID. Balanced crystalloids have an SID that is similar or higher than that of normal plasma (see Table 1) and will help to normalize SID in patients with a metabolic acidosis.

#### Lactate

Rapid administration of large volumes of lactate (L-lactate or a mixture of L and D-lactate) containing fluids can cause elevations in blood lactate. As lactate monitors used in clinical medicine only read L-lactate, the impact of lactate administration will be more evident with fluids containing higher levels of L-lactate. Healthy dogs receiving 180 ml/kg of LRS over an hour had a significant increase in blood lactate which was resolved 60 min after the end of the infusion (73). The increase in blood lactate following administration of lactate containing fluids is of a greater magnitude and persists for longer in hemodynamically unstable animals. Experimental studies exist comparing the administration of LRS vs. lactate free fluids in models of hemorrhagic shock (74, 75). The animals receiving LRS had a significant increase in blood lactate concentration following fluid resuscitation compared to the non-LRS animals. This was not associated with an acidosis because the lactate contained in IV fluids is a salt (sodium lactate) rather than lactic acid. This iatrogenic elevation of blood lactate can confuse the assessment of hemodynamic status.

The metabolism of lactate occurs primarily in the liver. Animals with significant hepatic dysfunction, such as hepatic failure or portosystemic shunt, may have limited lactate metabolism, resulting in elevations of blood lactate and lack of buffering capacity. For this reason, it is generally recommended to avoid lactate containing fluids in patients with liver dysfunction. Unlike lactate, acetate is readily metabolized by extra-hepatic tissues such as skeletal muscle and can be utilized by patients with hepatic dysfunction.

The infusion of lactate has been reported to have systemic effects including immune, coagulation and hemodynamic changes. Although there are a growing number of comparison studies between lactate and acetate containing fluids, the results are inconsistent, and more research is needed to fully understand these issues (76).

#### Dilutional Acidosis

A well-cited acid-base abnormality associated with fluid administration is a dilutional acidosis. Dilutional acidosis is the result of extracellular fluid volume expansion with a non-bicarbonate containing fluid (77). As a result, there is the same quantity of bicarbonate in a larger volume, leading to a fall in bicarbonate concentration. It has been most frequently associated with rapid infusions of large volumes of 0.9% sodium chloride. But it will occur with the infusion of any non-bicarbonate containing fluid such as LRS or Plasma-Lyte 148. This was demonstrated by a study in human cardiopulmonary bypass patients where they compared the use of Plasma-Lyte 148 with a buffer free fluid like 0.9% sodium chloride for initial pump prime (78). After 2 min of full pump flow both groups of patients had developed a similar degree of metabolic acidosis (dilutional acidosis), compared to baseline. Although at the end of the procedure the group that received Plasma-Lyte 148 had resolved their metabolic acidosis while the group with non-buffered fluids had a residual metabolic acidosis. The organic anions in balanced crystalloids help resolve the dilutional acidosis more rapidly, while patients receiving 0.9% sodium chloride take longer to resolve the acidosis. It is important to note that dilutional acidosis only occurs after rapid administration of exceptionally large volumes of fluids.

### Fluid Osmolality

Osmolality is a measure of the total concentration of solute (number of particles) in a quantity of solvent (number of osmoles/kg solution) and osmolarity is the number of osmoles/L of solution. In most clinical scenarios the mOsm/L is equivalent to the mOsm/kg, and the terms are considered interchangeable (79). When predicting the solute concentration of a solution through calculations adding together concentrations of known osmoles, it is the mOsm/L (osmolarity) that is being determined, whereas measured values (determined by freezing point depression) are reported as mOsm/kg (osmolality). This has some relevance to IV fluid solutions as the theoretical osmolarity tends to overestimate the measured osmolality because molecules do not always fully dissociate under physiologic conditions. For example 0.9% sodium chloride has a calculated osmolarity of 308 mOsm/L but the measured osmolality is 286 mOsm/kg (80).

The osmolality of an IV fluid can affect cells at the site of infusion before the fluid is diluted by the blood volume. Hyperosmolar fluids are of particular concern although very hypoosmolar fluids, such as sterile water, can also create injury. Peripheral vessels are small, and infused fluids have contact with endothelial cells in addition to erythrocytes. To avoid phlebitis, infusion of hyperosmolar fluids through a central vessel, where flow rates are faster, and the infused fluid is less likely to contact the vessel wall, is recommended. Studies suggest that a fluid osmolality >600 mOsm/L is too high for peripheral infusion (81, 82). A study of infusion of a parenteral nutrition solution with an osmolality of 840 mOsm/L via a peripheral vein in experimental dogs reported a high rate of thrombophlebitis at 36 h, which further supports this recommendation (83).

### Galactomannan

Plasma-Lyte 148 contains sodium gluconate that is produced from fermentation by Aspergillus sp., and it has been shown that the fluid can be contaminated by small quantities of galactomannan. Galactomannan is a component of the cell wall of Aspergillus, and galactomannan assays are used as diagnostic tests to identify aspergillus. The presence of Plasma-Lyte 148 in a diagnostic sample can cause a false positive result (84).

### Fluids for Medication Delivery

Any crystalloid can be used as a carrier or diluent for medications, and carrier fluids are typically either D5W or 0.9% sodium chloride, since these are not complex, are acidic, and are less likely to alter the medication it is carrying. The prescribing information of the individual drug to be infused will indicate which crystalloid carrier solution can be used. Balanced isotonic crystalloids can be co-administered with many medications, but some important drug incompatibilities have been identified. For example, Plasma-Lyte 148 is incompatible with pantoprazole, cyclosporine, and midazolam while LRS is incompatible with diazepam, ketamine, and cyclosporine (63, 85).

### Summary

The ideal balanced isotonic solutions will vary according to species. For most small animal patients, the traditionally coined “balanced” isotonic crystalloids are appropriate for fluid resuscitation, rehydration, and maintenance fluid support. In critically ill animals or patients with electrolyte or acid-base abnormalities and/or with hepatic disease, fluid choice should be based on the patient's existing acid/base and electrolyte condition, in consideration of the available information. Animals with extreme electrolyte imbalances should have a fluid prescription that corrects those imbalances without causing additional complications. Animals receiving high volumes of fluid therapy or several days of fluid therapy should be monitored for electrolyte and acid-base changes that could require a change in the fluid prescription. Hypoproteinemic animals may also benefit from infusion of a colloid solution.

## Author Contributions

All authors contributed equally to the research and writing of this manuscript.

## Conflict of Interest

The authors declare that the research was conducted in the absence of any commercial or financial relationships that could be construed as a potential conflict of interest.
