# Erratum: Crystalloid and Colloid Compositions and Their Impact

**DOI:** 10.3389/fvets.2022.878702

**Published:** 2022-03-17

**Authors:** Frontiers Production Office

**Affiliations:** Frontiers Media SA, Lausanne, Switzerland

**Keywords:** crystalloid, colloid, plasma, osmolarity, fluid therapy, COP, SID, Strong ion difference

Due to a production error, comments for the editor were erroneously published as part of the original article. The following has been removed:

“**Introduction**, paragraph 2, “(editor-See article 2 Advances in the Starling principle and Extracellular Matrix-Compliance?),” “(editor- See article Volume Kinetics?).”

**Fluid Types**, “*Hypertonic Crystalloids*,” paragraph 2, “(editor-see articles 10 and 11 Fluid resuscitation in critical ill non-hemorrhage hypovolemic animals and What are the dose response effects of IV fluids following hemorrhage and hemorrhage and trauma?)” and **paragraph 3**, “(editor- see article 12 on TBI?).”

**Colloid Solutions**, “*Synthetic Colloids*,” paragraph 2, “(editor- See Article 7 Colloids, Yes or No? Pros and Cons of Colloids?)” and paragraph 4, “(editor- See Article 7 Colloids, Yes or No? Pros and Cons of Colloids?).”

**Fluid Choice**, “*Sodium Concentration*,” paragraph 1, “(editor- see article 12 TBI?).”

The publishers apologize for this mistake. The original article has been updated.

## Publisher's Note

All claims expressed in this article are solely those of the authors and do not necessarily represent those of their affiliated organizations, or those of the publisher, the editors and the reviewers. Any product that may be evaluated in this article, or claim that may be made by its manufacturer, is not guaranteed or endorsed by the publisher.

